# Real-Time Imaging and Quantification of Amyloid-β Peptide Aggregates by Novel Quantum-Dot Nanoprobes

**DOI:** 10.1371/journal.pone.0008492

**Published:** 2009-12-30

**Authors:** Kiyotaka Tokuraku, Meg Marquardt, Tsuneya Ikezu

**Affiliations:** 1 Department of Pharmacology and Experimental Neuroscience, University of Nebraska Medical Center, Omaha, Nebraska, United States of America; 2 Department of Chemical Science and Engineering, Miyakonojo National College of Technology, Miyakonojo, Japan; Monash University, Australia

## Abstract

**Background:**

Protein aggregation plays a major role in the pathogenesis of neurodegenerative disorders, such as Alzheimer's disease. However, direct real-time imaging of protein aggregation, including oligomerization and fibrillization, has never been achieved. Here we demonstrate the preparation of fluorescent semiconductor nanocrystal (quantum dot; QD)-labeled amyloid-β peptide (QDAβ) and its advanced applications.

**Methodology/Principal Findings:**

The QDAβ construct retained Aβ oligomer-forming ability, and the sizes of these oligomers could be estimated from the relative fluorescence intensities of the imaged spots. Both QDAβ coaggregation with intact Aβ42 and insertion into fibrils were detected by fluorescence microscopy. The coaggregation process was observed by real-time 3D imaging using slit-scanning confocal microscopy, which showed a typical sigmoid curve with 1.5 h in the lag-time and 12 h until saturation. Inhibition of coaggregation using an anti-Aβ antibody can be observed as 3D images on a microscopic scale. Microglia ingested monomeric QDAβ more significantly than oligomeric QDAβ, and the ingested QDAβ was mainly accumulated in the lysosome.

**Conclusions/Significance:**

These data demonstrate that QDAβ is a novel nanoprobe for studying Aβ oligomerization and fibrillization in multiple modalities and may be applicable for high-throughput drug screening systems.

## Introduction

Neurodegenerative disorders such as Alzheimer's disease (AD), Parkinson's disease, Huntington's disease, and prion diseases are characterized by misfolded protein aggregates, termed amyloids, which are usually high in β-sheet content [1]. However, the exact mechanism of amyloid aggregation and its links to multiple disease pathogeneses are not fully understood. Amyloid-β peptide (Aβ) is the major component of senile plaques and is a hallmark of AD [2]. An early hypothesis stated that the accumulation of fibrillar Aβ deposits in senile plaques was neurotoxic [3]. In contrast, recent studies have identified the smaller soluble Aβ oligomer as potentially more neurotoxic than amyloid fibrils [4], [5], [6]. Meanwhile, Aβ peptide has been observed in various cellular localities, including lysosomes, aggresomes, mitochondria, dendritic spines, and within neurons, microglia, astrocytes and the extra-cellular space [7], [8], [9], [10], [11], but the exact cellular origin of Aβ aggregation is not known. To understand the mechanism of Aβ misfolding and locate the origin of Aβ assemblage, we have developed a real-time imaging tool for monitoring Aβ aggregation.

Fluorescent semiconductor nanocrystals (quantum dots; QD) have evolved over the past decade as highly useful fluorescence probes in biological staining and diagnostics [12], [13]. QD properties include long-term photostability, chemical and physical stability, nano-scale size, and multicolor fluorescence emission with single excitation [14]. These features are extremely useful for long-term, single-molecule imaging *in vitro* and *in vivo* [15], [16]. In fact, a single QD can be observed and tracked using basic wide-field fluorescence microscopy [17], confocal microscopy [12], total internal reflection microscopy [18], and two-photon fluorescent microscopy [19]. For these reasons, QD could be an excellent tool for real-time monitoring of Aβ aggregation and localization. Nevertheless, there have been no reports of successful preparation and characterization of QD-crosslinked Aβ peptide, possibly due to the difficulty of covalently coupling the QD to the peptide without also reducing the ability of Aβ to aggregate. Recently, Ji *et al.* [20] imaged Aβ42 and Aβ40 fibrils linked with QD, although the labeling was performed by non-specific ionic interaction between the fibrils and the QD. Therefore, the method is not applicable for tissue culture or *in vivo* studies. While fluorescein-labeled Aβ peptides have also been used in amyloid aggregation studies [21], [22], this application is limited to short-term live imaging studies (less than 1 second) and is not appropriate for small oligomer imaging as fluorescein is not suitable for single molecule imaging nor live imaging due to poor signal levels and quenching [23]. In addition, standard amyloid plaque staining by thioflavin or Congo red is not suitable due to poor binding between the fluorescent dyes and β-sheet structures of Aβ oligomers. Although potential cytotoxicity is a concern for long-term QD applications in cells [24], masking the core surface cadmium atom with a polyethylene glycol (PEG) coating greatly reduced the cytotoxicity [25]. Here, we have successfully generated a PEG-QD-crosslinked Aβ peptide, which has enabled us to quantitatively examine, for the first time, Aβ fibril and oligomer formations *in vitro* and in an intact cell system.

## Results

### Generation of QDAβ probe

Our first step was to examine whether Aβ42 or Aβ40 is more suitable as a QD probe. Both can be major components of amyloid plaques [2]. Aggregation of Aβ42 has been shown to be more rapid than Aβ40 [26]. Indeed, we confirmed that Aβ42, without SDS, begins to aggregate within minutes of preparation—already forming oligomers or protofibrils during the one hour labeling process (Fig. S1a). It formed trimeric and tetrameric species within 0.3 h from the start of incubation. On the other hand, Aβ40, without SDS, did not form oligomers after 5 days (Fig. S1c and S1d). Consequently, to provide a reasonable timeframe over which to study aggregation, we employed Aβ40 for QD-labeling in this study.

**Figure 1:**
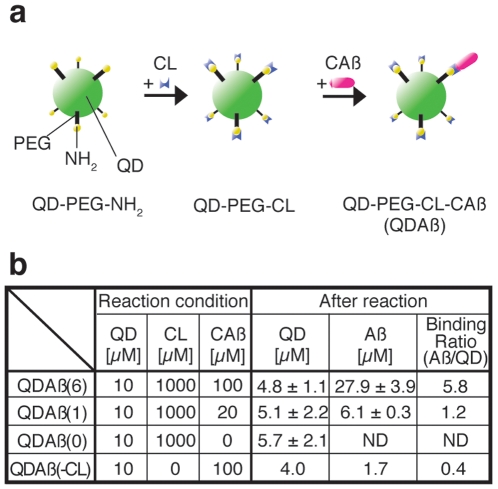
Preparation of QDAβ. (a) Scheme of QDAβ preparation. QD-PEG-NH_2_ was mixed with crosslinker (CL) and incubated for 1 h at 22°C. After elimination of unreacted CL by a desalting column, the intact QD-PEG-CL was mixed with various concentrations of Cys-Aβ40 (CAβ) and incubated for 1 h at 22°C. The maleimide group of CL was quenched by 2-mercaptoethanol, and unreacted CAβ was removed by desalting columns. (b) Yield of QDAβ preparation. The table shows reaction conditions and the yields of QD and Aβ.

Since Aβ is significantly smaller than any currently developed QDs, we also carefully considered the QD sizes and linker (between QD and Aβ) lengths. Here, we adopted polyethylene glycol (PEG)-conjugated Qdot 525 (QD-PEG-NH_2_), which is the smallest commercially available-QD with a 2,000 MW PEG linker. The QD-PEG-NH_2_ (10 µM) was first mixed with a cross-linker (CL), N-(6-maleimidocaproyloxy) sulfosuccinimide ester (Sulfo-EMCS, 1000 µM), to generate QD-PEG-CL (Fig. 1a). Since one QD-PEG-NH_2_ has ∼80 to 100 amine groups on the surface, CL saturates almost all PEG amino groups under these conditions. Next, 100, 20, and 0 µM Cys-Aβ40 (CAβ) was added to QD-PEG-CL, yielding the binding ratios (Aβ/QD) of 6, 1, and 0, for QDAβ(6), QDAβ(1), and QDAβ(0) (Aβ unconjugated control) conjugates, respectively (Fig. 1b). The initial concentration of Cys-Aβ40 could control the Aβ/QD ratio. A study to determine the rate of nonspecific binding between Cys-Aβ40 and non-crosslinked QD-PEG-NH_2_ showed an Aβ/QD binding ratio of 0.4, suggesting that approximately 7% of Cys-Aβ40 was bound to the QD surface via non-specific binding (Fig. 1b). The yield of QD particles was approximately 50% using this preparative method.

### Oligomer formation of QDAβ

Many recent studies have implicated soluble Aβ oligomers as a potential toxic species in AD pathology [4], [5], [6]. Since formation of the toxic, β-sheet rich, Aβ oligomer can be enhanced by certain concentrations of SDS [5], [6], we examined whether QDAβ forms oligomers in the presence or absence of SDS. Prior to conducting this experiment, we needed to confirm that 1 mM SDS enhances oligomerization of unlabeled Aβ40 and Aβ42 [5] by measuring the kinetics of Aβ40 and Aβ42 oligomerization with and without SDS (Fig. S1). SDS promoted oligomer and fibril formations of both Aβ42 and Aβ40 at 1 mM concentration with especially enhanced Aβ40 dimer formation (Fig. S1d). We then applied these conditions for monitoring oligomerization of QDAβ.

**Figure 2:**
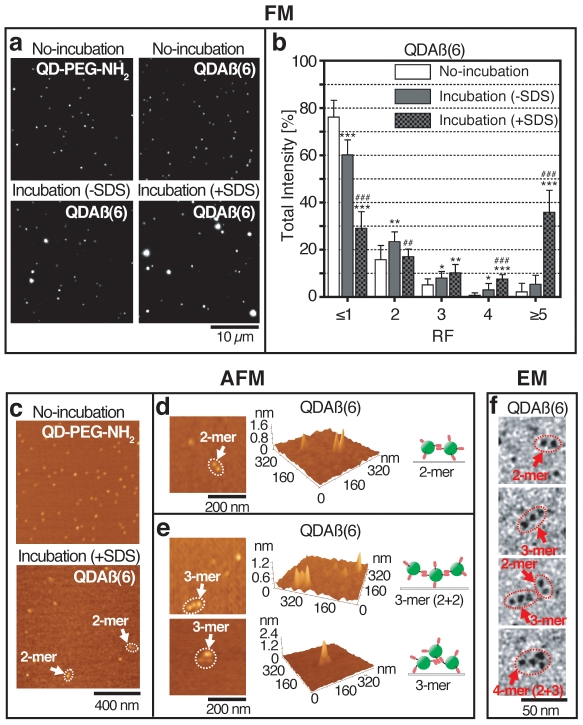
Imaging of QDAβ oligomers. (a) QD-PEG-NH_2_ (top left), non-incubated-QDAβ(6) (top right: stored in water for 3 weeks on ice), incubated-QDAβ(6) without SDS (bottom left: in PBS for 1 day at 37°C), and incubated-QDAβ(6) with SDS (bottom right: in PBS with 1 mM SDS for 1 day at 37°C) were observed by regular fluorescence microscopy using a 100x objective lens with QD filter set. (b) Distribution of QDAβ molecules belonging to each RF classes. The frequency of total intensity shows the sum of RF values of all spots belonging to the RF class. Error bars indicate standard deviation (SD, n = 10). * (or ^#^), ** (or ^##^), and *** (or ^###^) denote 0.01<*P*<0.05, 0.001<*P*<0.01, and *P*<0.001, respectively. * are non-incubated- versus incubated-samples (−SDS or +SDS) and ^#^ are -SDS versus +SDS samples. (c) QD-PEG-NH_2_ and incubated-QDAβ(6) with SDS under the same conditions as in (a) were observed by AFM. (d and e) Typical AFM images of dimeric (d) and trimeric (e) species. Trimers were classified into two types: two dimers (top, 2+2) or one trimer (bottom) of Aβ peptides. (f) Typical images of dimers, trimera, and tetramers by electron microscopy observations.

Oligomerization of QDAβ was imaged according to the method in Figure S2a. Incubation of QDAβ(6) in water for 3 weeks on ice does not alter its fluorescent image (Fig. 2a top right), suggesting that QDAβ(6) can be stored in water on ice without aggregation. In contrast, brighter and larger spots were observed by incubation of QDAβ(6) at 37°C with and without 1 mM SDS (Fig. 2a bottom micrographs), suggesting its oligomerization.

To examine the formation of oligomers by QDAβ, we measured the relative fluorescence (RF) and the number of fluorescence spots using the “analyze particle” tool of ImageJ (NIH) (Fig. S2b). In this analysis, the average RF of unconjugated QD-PEG-NH_2_ was expressed as 1 RF unit (RF1). Since fluorescence intensity is generally proportional to the number of fluorescence molecules, it is likely that the summed RF values indicate the total number of QDAβ molecules in each RF class. Therefore, we tallied the RF values for each RF class as total QDAβ spot intensity (RF≤1 to ≥5, Fig S2c). The data established that the distribution profile, as determined by the total intensity, of incubated-QDAβ(6) in water for 3 weeks on ice was similar to that of the negative control QD-PEG-NH_2_ (Table S1a). The results of incubation in the presence of SDS revealed that the percentage of QDAβ(6) molecules in the RF≤1 class were reduced from 76.2% to 29.1% after 24 hrs incubation at 37°C (Fig. 2b and Table S1b), suggesting that majority of QDAβ(6) particles formed oligomers in this condition. Although the oligomer formation was also observed with QDAβ(6) samples in the absence of SDS, the total value of RF2–RF≥5 (39.8%, Table S1c) was much less than in the presence of SDS (70.9%, Table S1b) (Fig. 2b). This enhancement of Aβ aggregation by SDS is consistent with the results obtained using unconjugated Aβ40 peptides (Fig. S1 b and d).

We also examined the effects of the Aβ/QD labeling ratio, in conjunction with SDS, on QDAβ oligomerization. In the presence of SDS, the frequency of spots belonging to the RF≤1 class significantly decreased as the Aβ/QD ratio increased (QDAβ(0)>QDAβ(1)>QDAβ(6)) (Table S1b). Accordingly, the number of spots in RF3, RF4, RF≥5 classes significantly increased in the order of QDAβ(0)<QDAβ(1)<QDAβ(6) (Table S1b). These data demonstrated that the Aβ/QD binding ratio is correlated with oligomer formation.

To confirm whether the bright, large spots were QDAβ oligomers, the incubated samples were examined by atomic force microscopy (AFM). The results revealed that several types of QD clustering were observed in QDAβ(6) but not in QD-PEG-NH_2_ samples (Fig. 2c). AFM imaging also revealed two types of trimers: one type entails a tandem repeat of three QDAβs (Fig. 2e, top), and another contains a triangular complex of three QDAβs (Fig. 2e, bottom). The distribution of oligomers that were measured from the AFM data (Table S2b) was similar to the fluorescence spot data (Table S2a) of the small oligomers (monomer, dimer, and trimer), suggesting that these small oligomer sizes can be estimated from RF values of fluorescence spots in fluorescence micrographs. We have also detected QDAβ oligomer formation as dimer, trimer, or tetramer by electron microscopy (Fig. 2f).

### Amyloid fibril formation with QDAβ

**Figure 3:**
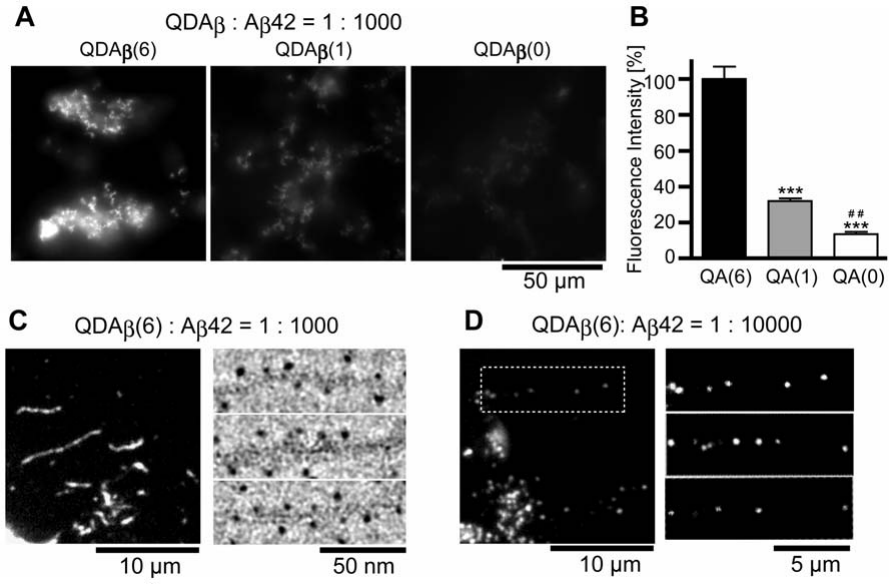
Coaggregation of intact Aβ42 and QDAβ. (a) 0.1% QDAβ(6)- (left), QDAβ(1)- (middle), and QDAβ(0)- (right) containing Aβ42 (final concentration 50 µM) were incubated in 96-well glass bottom plates at 37°C for 1 day, and observed by wide-field fluorescence microscopy using a 100x objective lens with FITC filter set. (b) Fluorescence intensities of the aggregates in (a). Fluorescence intensities measured from 20 randomly selected fields (100×100 pixel: 8.6×8.6 µm) containing the aggregates are shown as relative % intensity against QDAβ(6) group. *** denotes *P*<0.001 for QDAβ (6)- versus QDAβ (1)- or QDAβ (0)-samples and ## denotes 0.001<*P*<0.01 for QDAβ (1)- versus QDAβ (0) samples. Error bars indicate SD (n = 20). (c) 0.1% QDAβ-containing Aβ42 (final concentration 50 µM). Samples were incubated in microcentrifuge tubes at 37°C for 1 day, spread between glass slides and cover slips, and observed by regular microscopy using a 100x objective lens with QD filter set (left micrograph) and electron microscopy (right three micrographs). (d) 0.01% QDAβ-containing Aβ42 (final concentration 50 µM) were incubated in microcentrifuge tubes at 37°C for 1 day, spread between glass slides and cover slips, and observed by regular microscopy using a 100x objective lens with QD filter (left micrograph). The right three micrographs are magnified and brightened micrographs. The top right micrograph is the boxed area in the left micrograph.

Although we observed oligomerization solely between QDAβ particles, we were unable to observe amyloid fibril formation by QDAβ alone. This was expected because the size of the QD is significantly larger than that of Aβ. In fact, recent structural work on Aβ fibrils have revealed a non-registered parallel β-sheet structure stacking approximately 4 peptide molecules in 1 nm fibril length [27]. This implies that fibril formation is inhibited due to steric hindrance by the QDs. Therefore, unconjugated Aβ has to be incorporated with QDAβ to effectively image Aβ fibril formation. In this study, a QDAβ∶Aβ42 ratio of 1∶1000 (0.1%) or 1∶10000 (0.01%) was examined for Aβ aggregation (Fig. 3). When 0.1% QDAβ(6) was mixed with 50 µM Aβ42 peptide, bright aggregates were observed (Fig. 3a). The aggregates were stained with a monofluoro bis-styrylbenzene (FSB) derivative [28] (Fig. S3), demonstrating that these aggregates contain a β-sheet structure. Aggregates were also observed when 0.1% QDAβ(1) was mixed with 50 µM Aβ42. However, the mean fluorescence intensity of these aggregates was approximately 32% of that of QDAβ(6) (Fig. 3b), indicating that the insertion efficiency of QDAβ(1) into Aβ fibrils is lower than that of QDAβ(6). Although Aβ aggregates could also be visualized by incubation with 0.1% QDAβ(0), the fluorescence intensity was only approximately 14% of that of QDAβ(6) (Fig. 3b). This is probably due to non-specific binding between QD-PEG-NH_2_ and Aβ fibrils, as observed during the preparation of QDAβ(Fig. 1b) and in a previous report [20]. Individual Aβ filaments can be observed by high-power magnification (Fig. 3c left). Electron microscopy imaging revealed periodical insertion of QDAβ(6) into Aβ fibrils (Fig. 3c right). When 0.01% QDAβ(6) was incubated with Aβ42, the periodicity of single QD molecules was directly observed by fluorescent microscopy (Fig. 3d). The average interval length of the periodicity was 1.9±1.0 µm, which is close to the estimated value (2.5 µm) based on the NMR fibril structure [27]. These data suggest that QDAβ(6) was incorporated into Aβ fibrils with a similar efficiency as unconjugated Aβ42.

When various concentrations of Aβ42 containing 0.1% QDAβ(6) were incubated, time- and dose- dependent aggregation was observed (Fig. S4). No fibrils were observed in the sample of 6.3 µM Aβ42, suggesting that critical concentration of Aβ42 in fibril formation is between 6.3 and 13 µM. In addition, the coaggregation process with unconjugated Aβ42 was temperature-dependent, as we did not observe aggregation 1 day after incubation on ice (Fig. S5). This enables us to examine the aggregation process by live imaging system in a time-controlled manner.

### 4D imaging of Aβ aggregation *in vitro*

**Figure 4:**
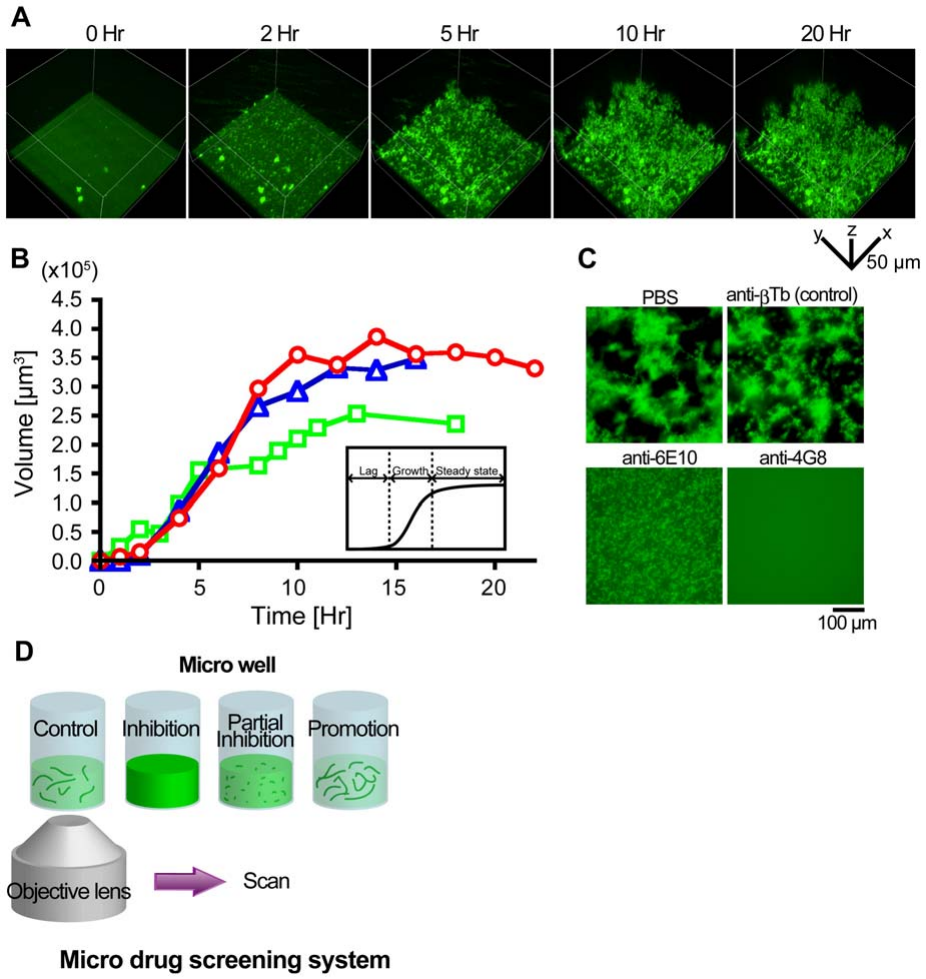
Imaging of Aβ aggregation *in vitro*. (a) 4D imaging of Aβ aggregation. 0.1% QDAβ(6)-containing Aβ42 (final concentration 100 µM) were incubated in a 96-well glass bottom plate at 37°C with controlled humidity and CO_2_ concentration, and observed by confocal microscopy every 30 min over 20 h using a 488 nm excitation laser (20% power) and a 60x oil objective lens. (b) Time-course of Aβ aggregation. The volumes of Aβ aggregates measured from three 4D experiments (red, blue, and green). The total volume of the observed space is 1.9×10^6^ µm^3^ (138×138×100 µm). The inset shows an idealized kinetic curve for amyloid aggregation [29]. Amyloid aggregation consists of lag, growth, and steady state phases. (c) Inhibition of Aβ aggregation by anti-Aβ antibody. 0.1% QDAβ(6)-containing Aβ42 (final concentration 13 µM) were incubated in PBS with 0.6 µM control antibody (anti-β ~tubulin, βTb; top right), 0.6 µM anti-6E10 antibody (bottom left), and 0.6 µM anti-4G8 antibody (bottom right) in a 96-well glass bottom plate at 37°C for 21 h, and observed by wide-field fluorescence microscopy. (d) The model of a micro drug screening system.

Since we succeeded in direct imaging of Aβ aggregation under a regular wide-field fluorescent microscope, we next improved the image quality by conducting time-dependent 3D imaging (4D imaging) of Aβ aggregation using automated Z-stack image acquisition of a Swept-field confocal microscope (Fig. 4a and Movie S1). When 0.1% QDAβ(6)-containing 100 µM Aβ42 was incubated at 37°C, small aggregates were observed on the glass bottom of the well within 1–2 h incubation time. The Aβ fibrils then “grew upwards” as Aβ that aggregated in solution precipitated on top of the fibrils (Movie S1 and S2). The time-course of the Aβ aggregation showed a typical sigmoidal curve [29] which consisted of the characteristic time lag, growth, and steady state phases (Fig. 4b). The lag time was approximately 1.5 h, and the aggregation reached a plateau around 12 h. Aggregation of Aβ can also be monitored by turbidity at 400 nm [26] and fluorescence measurement of thioflavin T (ThT) binding [5]. Turbidity measurements showed that the aggregation of 20 µM Aβ42 reached a plateau around 10–20 h in phosphate buffer (pH 7.4) [26] which is consistent with our 4D imaging in this study (Fig. 4A). In contrast, ThT binding of 10–35 µM Aβ42 reached a plateau around 1–2 h in phosphate buffer (pH 7.5 or pH 7.4) with approximately several minutes of lag time [5]. Our data suggest that the ThT binding assay monitors development of β-structure in both protofibrils and fibrils, and that the turbidity assay monitors the amount of fibrils but not protofibrils, presumably because the protofibrils (∼5 nm) [30] are too small to induce turbidity at 400 nm. These data imply the existence of two rate-limits in Aβ aggregation: β-structure formation, which can be detected as lag time of fluorescence of ThT, and protofibrillization. Total time of β-structure formation and protofibrillization is displayed as the lag time of QDAβ 4D imaging. Since the critical concentration of Aβ42 aggregation is between 6.3 and 13 µM (Fig. S5), as described above, approximately 90% of Aβ formed aggregates in a steady state phase. On the basis of 4D imaging data, we estimated that the density of stacked-Aβ aggregates on the glass bottom in a steady state was 32+/−6 mg/ml.

Next, we examined whether inhibition of Aβ aggregation could be observed using this technique (Fig. 4c). When 0.6 µM (0.1 mg/ml) anti-β-tubulin (βTb) mouse monoclonal control antibody was mixed with 13 µM of 0.1% QDAβ(6) containing Aβ42, the fibril formation was unaffected. In contrast, when 0.6 µM anti-Aβ mouse monoclonal antibodies (6E10 and 4G8, specifically recognizing Aβ1-16 and Aβ18-22 epitopes, respectively) were incubated, fibril formation was significantly inhibited (Fig. 4c). The effects of inhibition differed depending on antibodies: 6E10 blocked fibril elongation but not small aggregate formation, whereas 4G8 completely blocked Aβ aggregation. Since the 4G8 epitope corresponds to the region that forms a β-structure [31], the binding of 4G8 to the region may directly block the aggregation, whereas 6E10 may affect higher-order Aβ aggregation. 3D reconstruction of the Swept-field confocal microscope images demonstrated clear differences in the depth and size of Aβ aggregation in the presence of different antibodies (Fig. S6 and Movie S3–S5).

Fortunately, this image acquisition does not require the fixation or immobilization procedures necessary for AFM and electron microscopic observation. Furthermore, the fibril formation can be observed at a microscopic scale with the use of a simple bio-incubator system placed on the microscope stage. Thus, this technology can be applied to micro-scale screening of inhibitory drugs for Aβ aggregation (Fig. 4d).

### Live imaging of Aβ in cells

**Figure 5:**
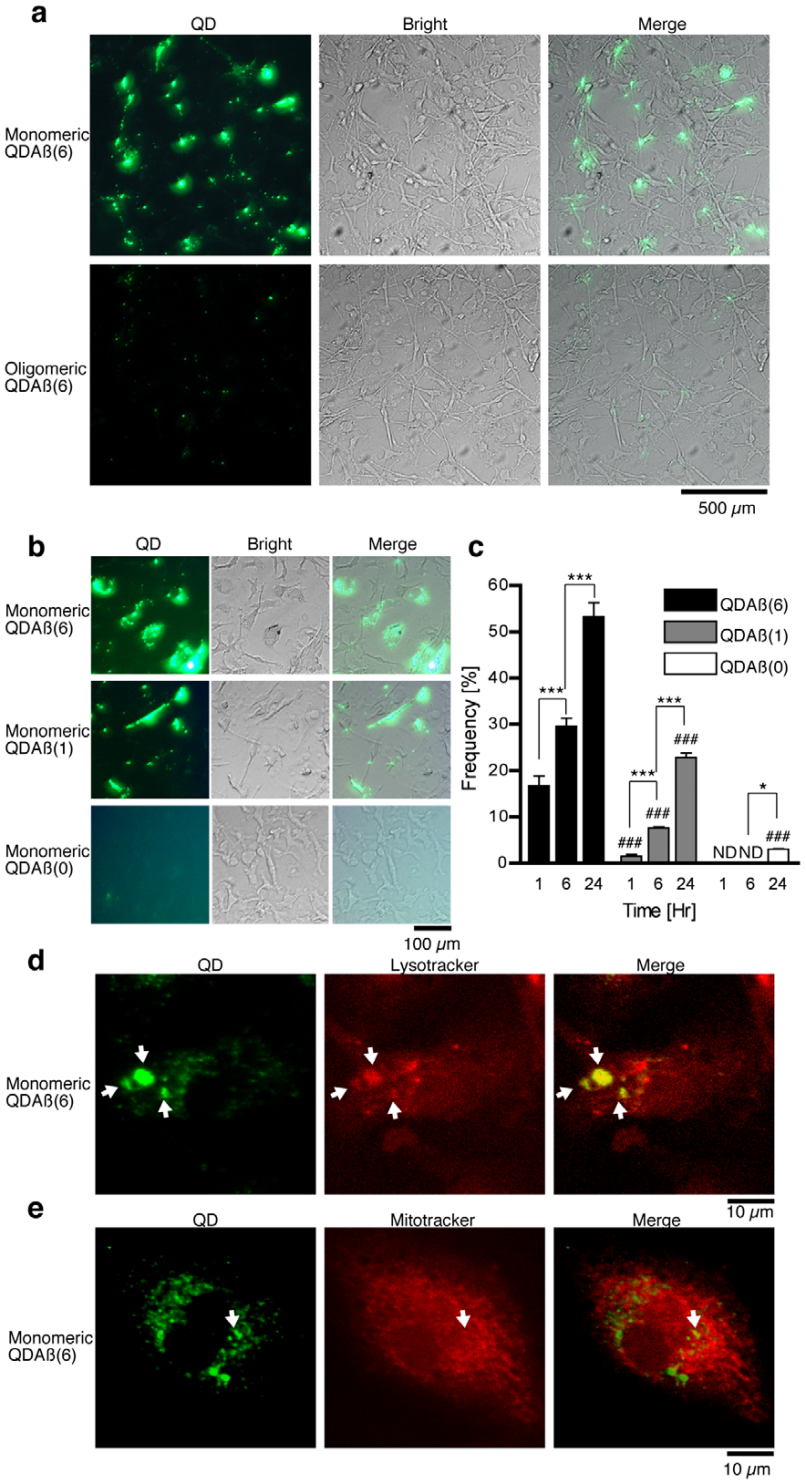
Imaging of QDAβ in cultured microglia. (a) Mouse microglia were incubated with 50 nM monomeric (top) or oligomeric (bottom) QDAβ(6) for 1 day, and observed by wide-field fluorescence microscopy using a 20x objective lens with FITC filter set. (b) Microglia were incubated with 50 nM monomeric QDAβ(6) (top), QDAβ(1) (middle), and QDAβ(0) (bottom) for 1 day, and observed by wide-field fluorescence microscopy using a 20x objective lens with FITC filter set. (c) Time-dependent monomeric QDAβ uptake by microglia. The number of cells with ingested QDAβ shown as the average percentage of total microglia. Error bars indicate SD (n = 3). ND: undetected. * and *** denote 0.01<*P*<0.05, and *P*<0.001, respectively, vs. 6 h time point of the same QD group, and ^###^ denotes *P*<0.001 vs. the same time point of QDAβ(6) samples. (d and e) Co-localization with Lysotracker (d) and Mitotracker (e). Microglia were incubated with 50 nM monomeric QDAβ(6) for 1 day, and then labeled with Lysotracker or Mitotracker, followed by confocal microscopy. Arrows show co-localization of QD (green) and Lysotracker/Mitotracker (red).

Microglia have been extensively shown to phagocytose Aβ [8], [9], [32]. Our recent study revealed that the uptake efficiency of the oligomeric Aβ was significantly lower (0.2-0.5%) than that of the monomeric form (1–10%) [9]. Therefore, we imaged Aβ phagocytosis by microglia using monomeric or oligomeric QDAβ (Fig. 5). When monomeric QDAβ(6) was incubated with primary cultured mouse microglia for 24 hr, QDAβ(6) uptake and accumulation was observed. In contrast, the uptake and accumulation of oligomeric QDAβ(6) was significantly less, supporting our recent finding [9]. When monomeric QDAβ(6) or QDAβ(1) was added to microglia (Fig. 5b), the number of cells containing phagocytosed material increased in a time-dependent manner (Fig. 5c and Fig. S7). In addition, the Aβ/QD ratio also affected the uptake rate as the amount of ingested QDAβ(6) was much higher than that of QDAβ(1). In contrast, uptake of QDAβ(0) was hardly observed under these conditions (Fig. 5b and c). Although high-power magnification imaging revealed that QDAβ(0) was also ingested by microglia, the ingestion amount was significantly lower than that of QDAβ(6) and QDAβ(1) (Fig. S8), indicating the ingestion and accumulation are due to Aβ peptides on the QD surface. There was no obvious cytotoxicity by ingestion of QD-probes, consistent with the report of the PEG-coated QD [25]. To determine the localization of the accumulated-QDAβ in microglia, we observed the uptake of monomeric QDAβ(6) using Lysotracker or Mitotracker. The results showed QDAβ(6) partially colocalized with lysosomes (Fig. 5d) as our group and others have reported [8], [9]. On the other hand, colocalization of QDAβ(6) and Mitotracker, although observed in a recent report [10], was less than that of Lysotracker (Fig. 5e). These results indicate that the majority of accumulated Aβ colocalized with lysosomes, but not with mitochondria. The colocalization of QDAβ(6) and Lysotracker was reconstructed in 3D-images (Fig. S9 and Movie S6).

## Discussion

In this study, we developed a method for QD-labeling of Aβ, which can then be utilized to monitor Aβ aggregation for real-time imaging both *in vitro* and in cells. We believe that this technology can be applied to a wide variety of amyloidogenic peptides and proteins.

Our study found that QDAβ forms oligomers and that small oligomer sizes can be estimated from fluorescent microscope imaging. In addition, we could also observe monomeric QDAβ uptake by microglia (Fig. 5), suggesting the application of QDAβ for the functional analysis of Aβ oligomers. Although the data *in vitro* and in cells showed that the properties of QDAβ oligomers are similar to those of untagged Aβ oligomers, it is still not known whether the structures of QDAβ oligomers and native Aβ oligomers are quite the same. Indeed, QDAβ failed to form fibrils by itself assumedly due to steric hindrance by the QDs, suggesting a possibility that this also precludes high molecular oligomer formation (such as nonamer or dodecamer). However, QDAβ is a useful nanoprobe, if used as a small fraction of the unmodified Abeta, for monitoring the aggregation under the microscope.

In this study, we also successfully observed quantitative 4D live imaging of Aβ aggregation (Fig. 4 a and b, Movie S1). Moreover, the inhibition of the Aβ aggregation by anti-Aβ antibody could be observed in 3D reconstructed imaging. This method could visualize a detailed configuration of Aβ aggregates at a microscopic scale, suggesting an application for advanced micro drug screening systems that can distinguish different inhibition mechanisms of Aβ aggregation at different stages.

In this study, we successfully observed different ingestion manners between monomeric and oligomeric QDAβ by microglia (Fig. 5). The lysosomal accumulation of oligomeric QDAβ was poorer than that of the monomeric form, suggesting that it is difficult for microglia to phagocytize oligomerc Aβ. These data imply that cytotoxic Aβ oligomers [4], [5] are less prone to degradation by microglia in the brain.

Since QD can be detected by multi-photon fluorescence microscopy [19], this technology could be applied to monitor localization and aggregation of Aβ in brain. Recently, it was reported that transferrin (Tf)-conjugated quantum rods transmigrated across an *in vitro* blood-brain barrier model via receptor-mediated transport [33]. If QDAβ can be coated with Tf and the nanoprobe retains the transmigration capability, it may become a powerful tool for *in vivo* live imaging of Aβ aggregation in the brain. Further development of QDAβ nanoprobes on the basis of this outcome promises to yield useful information in the analysis of beta-amyloidosis—a hallmark of AD.

## Materials and Methods

### Materials

Human amyloid peptides of Cys-Aβ40 (Anaspec), Aβ40 (Biosource), and Aβ42 (Biosource) were dissolved at a concentration of 1 mg/ml in 100% 1,1,1,3,3,3-hexafluoro-2-propanol (Acros), incubated at 22°C for 1 h, and sonicated for 10 min. The aliquots were put in microcentrifuge tubes, dried down, and stored at −20°C. N-(6-maleimidocaproyloxy) sulfosuccinimide ester (Sulfo-EMCS) (Pierce) was stored at 4°C and dissolved immediately before use. Qdot 525 ITK amino (PEG) quantum dot (QD-PEG-NH_2_) (Invitrogen) was stored at 4°C. Dulbecco's Phosphate-buffered saline (Mediatech) was used as PBS. All other chemicals were of reagent grade.

### Preparation of QDAβ

12.5 µl of 8 µMQD-PEG-NH_2_ (100 pmol) was put in microcentrifuge tubes and centrifuged at 10,000×g for 1 min at 4°C to eliminate any aggregates. The supernatants were transferred into centrifugal filter units (Microcon YM-100, Millipore), and the remaining volume of the unit was filled with 450 µl of PBS. After centrifugation at 2,800×g for 15 min at 4°C, the unit was refilled with 450 µl of PBS and centrifuged again at 4°C until the volume was reduced to 5 µl. The QD-PEG-NH_2_ solutions were adjusted to 9 µl with PBS, supplemented with 1 µl of 10 mM sulfo-EMCS (final concentrations: 10 µM QD-PEG-NH_2_, 1000 µM sulfo-EMCS, and PBS), and incubated for 1 h at 22°C. To quench unreacted sulfo-EMCS, the reacted samples (QD-PEG-CL) were supplemented with 1 µl of 100 mM K-glutamate (pH 7.4) and incubated for 10 min at 22°C. The buffer was changed by micro spin desalting columns (Zeba Micro Spin Desalting Column, Pierce) that were equilibrated with 5 mM EDTA in PBS (pH 6.8) (PBSE), and the volumes were adjusted to 9 µl with PBSE. Meanwhile, dried Cys-Aβ40 aliquots were dissolved at a concentration of 1000 or 200 µM in dimethyl sulfoxide (DMSO). The QD-PEG-CL solutions were mixed with various concentrations of Cys-Aβ40 (final concentration 100, 10, or 0 µM), and incubated for 1 h at 22°C. To quench the unreacted maleimide group of EMCS, 1 µl of 100 mM 2-mercaptoethanol was added and incubated for 10 min at 22°C. The buffer was changed to pure water using micro spin desalting columns. Concentrations of QD in QDAβ were determined at the absorbance of 504 nm according to the instruction manual (Invitrogen). Concentrations of Aβ40 in QDAβ were measured using Human β Amyloid 1–40 ELISA KIT (Biosource).

### Kinetic analysis of Aβ40 and Aβ42 aggregations by SDS-PAGE

Dried Aβ40 and Aβ42 aliquots were dissolved at a concentration of 1 mM in DMSO. These Aβ solutions were diluted at 50 µM in PBS with or without 1 mM SDS, and 5 µl of aliquots were incubated for various time periods at 37°C. The samples were mixed with sample buffer (final concentration: 50 mM Tris-HCl (pH 6.8), 2% SDS, 0.1% bromophenol blue, and 10% glycerol) and immediately electrophoresed using 16.5% Tris-Tricine gels [34] or 16% Tris-Glycine gels [35]. These gels were stained with Coomassie brilliant blue.

### Imaging of QDAβ oligomers

QDAβ samples were adjusted to a concentration of 3.0 µM in PBS with or without 1 mM SDS, and 5 µl aliquots were incubated for various time periods at 37°C. The oligomer samples were observed by wide-field fluorescence microscopy, atomic force microscopy (AFM), and electron microscopy. Details of sample preparation and analysis in fluorescence microscopy observations are provided in Supplementary Figure 2.

### Imaging of fibril formation using QDAβ

Dried Aβ42 aliquots were dissolved at various concentrations (100, 50, 25, 13, and 6.3 µM) in PBS and mixed with 0.1% or 0.01% QDAβ(6). To remove any aggregates, the mixtures were centrifuged at 10,000×*g* for 1 min at 4°C. The mixtures (50 µl) were put in microcentrifuge tubes or 96-well glass bottom plates (MatTek). The samples in microcentrifuge tubes were incubated for various time periods at 37°C in an air incubator, and observed by wide-field fluorescence microscopy or electron microscopy. The samples in 96-well glass bottom plates were incubated at 37°C in 5% CO_2_ in a culture chamber (LiveCell™, Pathology Devices), and directly observed by wide-field fluorescence microscopy or swept-field laser-scanning confocal microscopy [QDAβ: Excitation: 488 nm at 15% power; Emission filter: Chroma Quad Filter (#C68208) for FITC].

### Inhibition of fibril formation by anti-Aβ antibody

Aβ42 was dissolved in PBS and mixed with 0.1% QDAβ(6) (final concentrations: 16 nM QDAβ(6), and 16 µM Aβ42). 40 µl of the mixtures were mixed with 10 µl of 1 mg/ml control IgG (anti-β ~tubulin, G712A, Promega) or 1 mg/ml anti-Aβ IgG (anti-6E10, SIG-39320, Signet; anti-4G8, SIG-9240, Signet) (final concentrations: 0.1 mg/ml IgG, 13 µM Aβ42, and 13 nM QDAβ(6)), and centrifuged at 10,000×g for 1 min at 4°C to remove any aggregates. The supernatants were incubated in 96-well glass bottom plates for 1 day at 37°C, and observed with wide-field fluorescence microscopy or swept-field laser-scanning confocal microscopy.

### Primary culture of microglia

Microglia were prepared according to the previous report [36]. Microglia were prepared from wild type mouse day 0 newborn pups as described [37], and cultured in Dulbecco's modified eagle medium supplemented with heat-inactivated 10% fetal bovine serum, heat-inactivated 5% horse serum, and 50 µg/ml penicillin/streptomycin (all from Invitrogen). Microglia released in the tissue culture media by shaking were collected at 14 days after the plating. After confirmation of their purity to be more than 90% by immunocytochemistry (CD11b for microglia, GFAP staining for contaminating astrocytes, and Hoechst 33342 for nuclear staining), cells were used for experiments. The primary cultures were cultured at 37°C in 5% CO_2_.

### Preparations of monomeric and oligomeric QDAβ for *in vivo* imaging

To prepare oligomeric QDAβ samples, QDAβ was adjusted to a concentration of 3.0 µM in PBS with 1 mM SDS, and then incubated for 1 day at 37°C. Monomeric QDAβ samples were identical but were not incubated.

### Uptake of QDAβ by microglia and imaging with Lysotracker and Mitotracker

Mouse microglia were seeded at a density of 50,000 cells/well in 96-well glass bottom plates and preincubated for 10 days. The cells were incubated with 50 nM monomeric or oligomeric QDAβ for 1 day, and then supplemented with 50 nM Lysotracker (Invitrogen) or 100 nM Mitotracker (Invitrogen). After an additional incubation for 30 min, the cells were fixed with 4% paraformaldehyde (PFA) for 15 min at 22°C and washed with PBS three times. Vectashield (Vector Laboratories) was added to the wells, and the cells were observed by wide-field fluorescence microscopy or swept-field confocal microscopy. [Lyso/Mitotracker: Excitation: 568 nm at 25% power; Emission filter: Chroma Quad Filter (#C68208) for Texas Red. QDAβ: Excitation: 488 nm at 20% power; Emission filter: Chroma Quad Filter (#C68208) for FITC].

### Wide-field fluorescence microscopy

The samples were observed with a wide-field fluorescence microscope (TE-300, Nikon) equipped with CCD camera (DP71, Olympus). QD was observed using a QD filter set (ex 405/20 and em 430LP; Chroma/Nikon) or FITC filter set (ex 480/40 and em 535/50, Chroma/Nikon). The FSB derivative, (*E,E*)-1,4-bis(4-hydroxy)styrylbenzene [28], was observed using a Blue filter set (ex 390/22 and em 460/50, Chroma/Nikon) (Blue).

### Atomic force microscopy

Reaction mixtures were deposited on a 1-(3-Aminopropyl) silatrane- (APS-) modified mica [38], [39] glued to the glass slide. AFM images were taken in air, height, amplitude and phase mode using MFP-3D Asylum Research Instrument (Santa Barbara, CA). Regular silicon probes (TESP) with spring constant 40 N/m and resonance frequencies 270–320 kHz were used. Image processing and the cross-section measurements were performed using Femtoscan (Advanced Technologies Center, Moscow, Russia).

### Electron microscopy

Reaction mixtures were spread on carbon-coated grids, negatively-stained with 2% uranium acetate pH 7.0, and examined under an electron microscope (H-7500, Hitachi) with an acceleration voltage of 75 kV as described [40]. Images were processed using the FFT bandpass filter (ImageJ 1.40 g, NIH).

### Swept-field laser-scanning confocal microscopy

Co-aggregation of QDAβ-Aβ42 and microglial cells in 96-well glass bottom plates was observed using a swept-field laser-scanning confocal microscopy system (TE-2000U, Nikon). QD was excited by an argon laser, and Lysotracker and Mitotracker were excited by an argon/krypton laser.

### Statistics

Data were analyzed by analysis of variances, followed by one-way ANOVA (Newman-Keuls multiple comparison tests) using statistics software (Prism 4.0, GraphPad Software inc.).

## Supporting Information

Table S1 Distribution of QDAβ molecules belonging to each RF class as determined by the total intensity of QDAβ. (a) Distribution of total fluorescent intensity (%) of unconjugated QD-PEG-NH2 and QDAβ(6). QD-PEG-NH2 in 50 mM borate was diluted with PBS (final 1-10 nM) and then analyzed immediately. QDAβ(6) samples (3.0 µM) in water, PBS, and PBS containing 1 mM SDS were incubated for 3 weeks at 0°C, for 6 weeks at 4°C, and for 3 weeks at 37°C, respectively. The samples were diluted with PBS (final 1-10 nM) and then analyzed immediately. (b and c) 3.0 µM QDAβ(0), QDAβ(1), and QDAβ(6) were incubated in PBS with (b) or without (c) 1 mM SDS for 1 day at 37°C. The samples were diluted with PBS (final 1–10 nM) and then analyzed immediately. The data show averages of 10 fields (86×86 µm). Incubation of QDAβ(6) in PBS for 6 weeks at 4°C led to a significant increase in the total value of the RF2-RF≥5 classes (23.8% to 70.3%) and a decrease in the RF≤1 class (76.2% to 29.7%), suggesting that QDAβ(6) forms oligomers in PBS at 4°C. In contrast, QDAβ(6) incubated in water for 3 weeks on ice was similar to that of the negative control QD-PEG-NH2, suggesting that QDAβ(6) can be stored in water on ice but not in PBS in the refrigerator. Distribution of QDAβ molecules belonging to each RF class as determined by the total intensity of QDAβ. (a) Distribution of total fluorescent intensity (%) of unconjugated QD-PEG-NH2 and QDAβ(6). QD-PEG-NH2 in 50 mM borate was diluted with PBS (final 1-10 nM) and then analyzed immediately. QDAβ(6) samples (3.0 µM) in water, PBS, and PBS containing 1 mM SDS were incubated for 3 weeks at 0°C, for 6 weeks at 4°C, and for 3 weeks at 37°C, respectively. The samples were diluted with PBS (final 1-10 nM) and then analyzed immediately. (b and c) 3.0 µM QDAβ(0), QDAβ(1), and QDAβ(6) were incubated in PBS with (b) or without (c) 1 mM SDS for 1 day at 37°C. The samples were diluted with PBS (final 1–10 nM) and then analyzed immediately. The data show averages of 10 fields (86×86 µm). Incubation of QDAβ(6) in PBS for 6 weeks at 4°C led to a significant increase in the total value of the RF2-RF≥5 classes (23.8% to 70.3%) and a decrease in the RF≤1 class (76.2% to 29.7%), suggesting that QDAβ(6) forms oligomers in PBS at 4°C. In contrast, QDAβ(6) incubated in water for 3 weeks on ice was similar to that of the negative control QD-PEG-NH2, suggesting that QDAβ(6) can be stored in water on ice but not in PBS in the refrigerator. Although longer incubation (3 weeks) showed a slight promotion of Aβ aggregation in the presence of 1 mM SDS (a, far right), the distribution profile was similar to the 1 day incubated sample (b, far right). These results revealed that oligomer formation of QDAβ(6) nearly saturates after 24 hrs, and that approximately 30% of QDAβ(6) remains as monomers under these conditions.
(0.05 MB DOC)

Table S2 Comparison of QDAβ comets as determined by fluorescence microscopy and AFM imaging. (a) Frequency of spot number belonging to each RF class from fluorescence microscope observations. The data table shows differences before (1) and after incubation (2). The data of RF≤1 (parenthetic data) alone were estimated according to the following calculation method because the RF≤1 value of (2) - (1) was not correct. RF≤1 value of (2) - (1) calculated by 100 - (RF2+RF3+RF4+RF≥5). (b) Frequency of multimerization from AFM observations. The data represent averages of 9 fields (1600×1600 nm). This comparison shows that the frequency of small oligomers (1-mer, 2-mer, and 3-mer) is similar to the frequency of RF values, suggesting that small oligomer sizes can be estimated from fluorescence intensities.
(0.04 MB DOC)

Figure S1 Kinetics of Aβ42 and Aβ40 aggregations. 50 µM Aβ42 peptide (a and b) and 50 µM Aβ40 peptide (c and d) were incubated in PBS with or without 1 mM SDS for various time periods at 37°C. After the incubation, these samples were electrophoresed using 16.5% Tris-Tricine [1] (a and c) and 16% Tris-Glycine gels [2] (b and d). Aggregation of Aβ42 was more rapid than Aβ40 in PBS both with and without SDS. [1] Schagger H (2006) Tricine-SDS-PAGE. Nat Protoc 1: 16–22. [2] Laemmli UK (1970) Cleavage of structural proteins during the assembly of the head of bacteriophage T4. Nature 227: 680–685.
(1.27 MB TIF)

Figure S2 Analysis of fluorescence spots of QDAβ oligomers. (a) Preparation of samples. The coverslips for wide-field fluorescence microscopy observation were prepared by the modified method of Agrawal et al. [3]. An aliquot (2 µl) of oligomer sample solution, which was diluted to 1–10 nM, was spread between the glass slide and the coverslip. The coverslip was taken off, dried, and placed on a wide-field fluorescence microscope. The gray images (2040 pixel×1536 pixel: 175 µm×132 µm) were obtained using a 100x objective lens with a QD filter set. A micrograph represented an average of 5 frames (each exposure time was 0.2 s). (b) Measurement of relative fluorescence. The micrographs were analyzed using ImageJ software (NIH). In this analysis, we used a 1000×1000 pixel area in the central region of the micrographs because of aberration at the periphery. The micrographs were thresholded under the same conditions and then were analyzed using the “analyze particles” program of ImageJ. Relative fluorescence (RF) was defined as the product of the area size (pixel) and mean fluorescence intensity. The average RF of unlabeled QD-PEG-NH2 was expressed as 1 RF unit (RF1). In this study, ≤1.5, 1.5–2.5, 2.5–3.5, 3.5–4.5, ≥4.5 of RF were indicated as RF≤1, RF2, RF3, RF4, and RF≥5, respectively. Each analysis averaged 10 micrographs (one micrograph contained several hundred particles). (c) Frequency of spot number and total fluorescent intensity. Spot number (Table S2) and total fluorescent intensity (Figure 2b and Table S1) reflect the number of oligomers and the number of QDs belonging to each RF class. [3] Agrawal A, Deo R, Wang GD, Wang MD, Nie S (2008) Nanometer-scale mapping and single-molecule detection with color-coded nanoparticle probes. Proc Natl Acad Sci U S A 105: 3298–3303.
(0.43 MB TIF)

Figure S3 Staining of Aβ coaggregates by FSB derivative, (E,E)-1,4-bis(4-hydroxy)styrylbenzene. 0.1% QDAβ(6)-containing Aβ42 (final concentrations: 50 nM QDAβ(6) and 50 µM Aβ42) were incubated in PBS without (a) or with (b) 1 µM FSB derivative (BHSB) for 1 day at 37°C in 96 well glass bottom plates (MatTek). The aggregates were observed by wide-field fluorescence microscopy using a 20x objective lens with FITC (QD) or Blue (Blue) filter sets. Since the FSB derivative binds to the β-sheet structure of Aβ fibrils [4], it is likely that these aggregates are typical Aβ fibrils containing β-sheet structure. [4] Flaherty DP, Walsh SM, Kiyota T, Dong Y, Ikezu T, et al. (2007) Polyfluorinated bis-styrylbenzene beta-amyloid plaque binding ligands. J Med Chem 50: 4986–4992.
(2.49 MB TIF)

Figure S4 Dose- and time-dependent coaggregation. 0.1% QDAβ(6)-containing Aβ42 (50, 25, 13, and 6.3 µM of Aβ42) were incubated at 37°C in 96 well glass bottom plates. The samples were observed at 0, 4, and 21 h from the start of incubation by wide-field fluorescence microscopy using a 20x objective lens with FITC filter set. No aggregates were observed in all 0 h samples. Although dose- and time-dependent aggregation were observed in the 50, 25, and 13 µM samples, aggregates were not observed in the 6.3 µM sample, suggesting that the critical concentration for Aβ42 aggregation was between 6.3–13 µM under these conditions.
(7.12 MB TIF)

Figure S5 Temperature-dependent Aβ aggregation. 0.1% QDAβ(6)-containing Aβ42 (final concentration 50 µM) was incubated for 1 day at 37°C (left), for 1 day on ice (middle), and for 1 day at 37°C after 1 day on ice (right), and observed by wide-field fluorescence microscopy using a 100x objective lens with FITC filter set. The results showed that Aβ aggregates were not formed after 1 day at 0°C incubation (middle). The sample on ice formed aggregates by additional incubation (right), suggesting that the 0.1% QDAβ(6)-containing Aβ42 mixture can be stored on ice for at least 1 day.
(1.67 MB TIF)

Figure S6 3D reconstruction images of Aβ aggregation inhibition by anti-Aβ antibody. 0.1% QDAβ(6)-containing Aβ42 (final concentration 13 µM) was incubated in PBS without antibody (left), with anti-βTubulin (anti-βTb) antibody (middle), and with anti-6E10 antibody (right) for 1 day at 37°C in 96 well glass bottom plates, and observed by swept-field laser-scanning confocal microscopy using a 488 nm excitation laser (75%) and a 100x objective lens. The movies of these 3D images are supplied in Movie S2-S4.
(3.87 MB TIF)

Figure S7 Microglial uptake of monomeric QDAβ(6), QDAβ(1), and QDAβ(0). Medium containing monomeric QDAβ(6) (a), QDAβ(1) (b), or QDAβ(0) (c) (final concentration 50 nM) was added to primary cultured mouse microglia in 96-well glass bottom plates (50,000 cells/well), and incubated for 1, 6, and 24 h time periods. The cells were fixed with 4% PFA and observed by wide-field fluorescence microscopy using a 20x objective lens with QD filter set.
(8.75 MB TIF)

Figure S8 Magnified observation of microglia with ingested monomeric QDAβ(6) and QDAβ(0). Primary mouse microglia were incubated with monomeric QDAβ(6) or QDAβ(0) (final concentration 50 nM) for 24 h, followed by fixation with 4% PFA, and observed by wide-field fluorescence microscopy using a 100x oil objective lens (TE-300, Nikon Instruments) and QD filter set (green).
(5.75 MB TIF)

Figure S9 Co-localization of ingested QDAβ6) and Lysotracker in microglia. Primary mouse microglia were incubated with 50 nM monomeric QDAβ6) for 24 h, followed by incubation with 50 µM Lysotracker for an additional 30 min. The cells were fixed with 4% PFA, and observed by Swept-field laser-scanning confocal microscopy using 488 nm excitation (QD, green) and 568 nm excitation (Lysotracker, red). Far right panel is the 3D reconstruction image in the same field. The movie of this 3D image is in Movie S5.
(3.22 MB TIF)

Movie S1 4D imaging of Aβ aggregation. 0.1% QDAβ(6)-containing Aβ42 (final concentration 100 µM) was incubated in PBS for 24 h at 37°C in 96 well glass bottom plate. The sample was observed every 30 min by swept-field confocal microscopy using 488 nm excitation laser (20%) and 60x objective lens. The bird's-eye movie is played at a speed of 4 h/s.
(5.68 MB MOV)

Movie S2 4D imaging of Aβ aggregation. 0.1% QDAβ(6)-containing Aβ42 (final concentration 100 µM) was incubated in PBS for 24 h at 37°C in 96 well glass bottom plate. The sample was observed every 30 min by swept-field confocal microscopy using a 488 nm excitation laser (20%) and 60x objective lens. The movie in angled bird's-eye view is played at a speed of 4 h/s.
(5.68 MB MOV)

Movie S3 3D imaging of Aβ aggregates in PBS. 0.1% QDAβ(6)-containing Aβ42 (final concentration 50 µM) was incubated in PBS for 1 day at 37°C in a 96 well glass bottom plate. The sample was observed by swept-field confocal microscopy using a 488 nm excitation laser (75%) and 100x objective lens.
(5.53 MB MOV)

Movie S4 3D imaging of Aβ aggregates in PBS with anti-βTubulin (control) antibody. 0.1% QDAβ(6)-containing Aβ42 (final concentration 50 µM) was incubated in PBS containing anti-βTubulin antibody for 1 day at 37°C in a 96 well glass bottom plate. The sample was observed by swept-field confocal microscopy using a 488 nm excitation laser (75%) and 100x objective lens.
(5.53 MB MOV)

Movie S5 3D imaging of Aβ aggregation in PBS with anti-6E10 antibody. 0.1% QDAβ(6)-containing Aβ42 (final concentration 50 µM) was incubated in PBS containing anti-6E10 antibody for 1-day at 37°C in a 96 well glass bottom plate. The sample was observed by swept-field confocal microscopy using a 488 nm excitation laser (75%) and 100x objective lens.
(5.53 MB MOV)

Movie S6 3D movies of QDAβ(6) and Lysotracker in Fig. S9 (far right panel).
(4.76 MB MOV)
