# Supplementary figures and images for: Real-Time Imaging and Quantification of Amyloid-β Peptide Aggregates by Novel Quantum-Dot Nanoprobes
Source: PLoS One. 2009 Dec 30;4(12):e8492. doi: 10.1371/journal.pone.0008492 (PMC2794548; doi:10.1371/journal.pone.0008492)

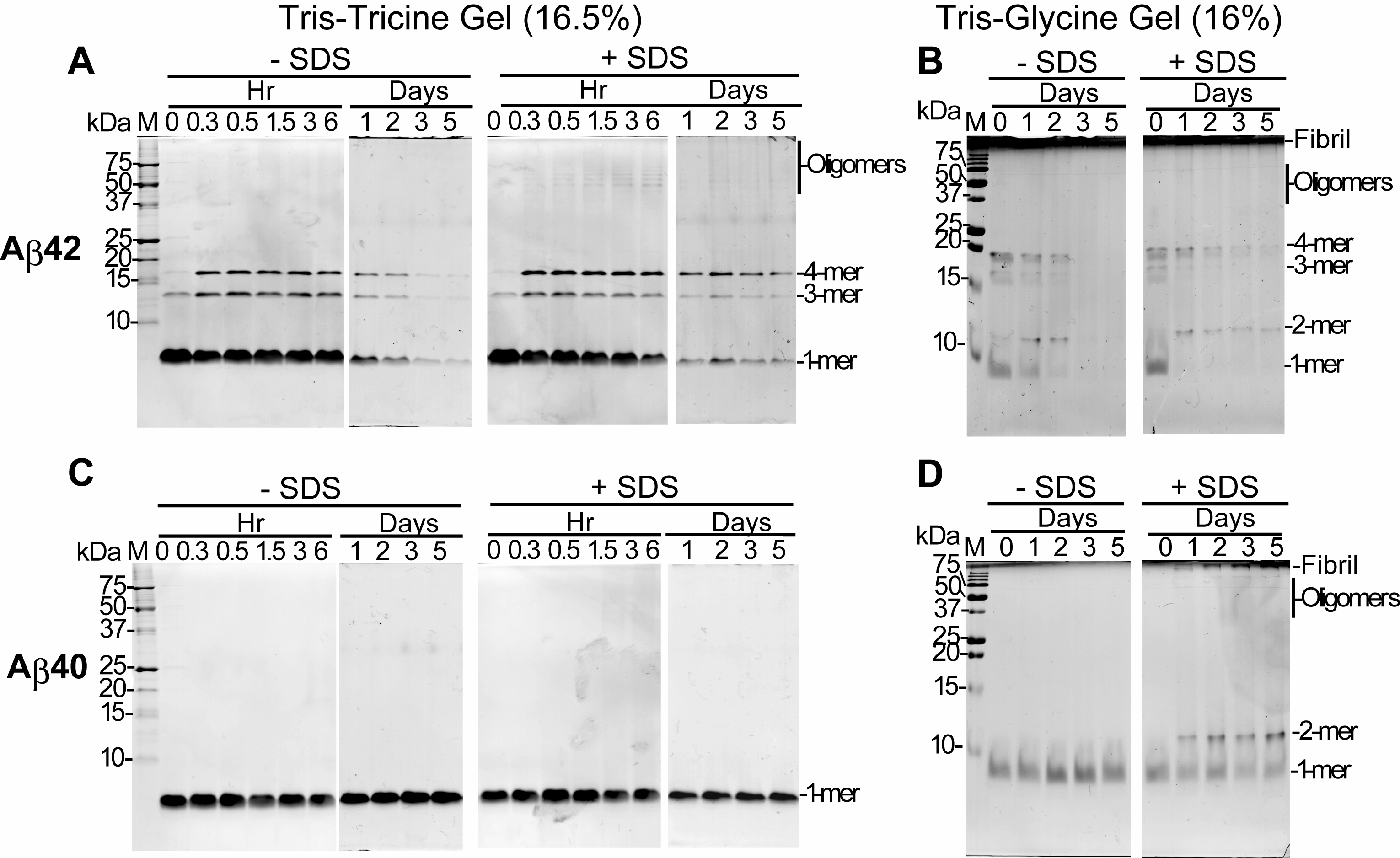

Supplement: Figure S1 — Kinetics of Aβ42 and Aβ40 aggregations. 50 µM Aβ42 peptide (a and b) and 50 µM Aβ40 peptide (c and d) were incubated in PBS with or without 1 mM SDS for various time periods at 37°C. After the incubation, these samples were electrophoresed using 16.5% Tris-Tricine [1] (a and c) and 16% Tris-Glycine gels [2] (b and d). Aggregation of Aβ42 was more rapid than Aβ40 in PBS both with and without SDS. [1] Schagger H (2006) Tricine-SDS-PAGE. Nat Protoc 1: 16–22. [2] Laemmli UK (1970) Cleavage of structural proteins during the assembly of the head of bacteriophage T4. Nature 227: 680–685. (1.27 MB TIF) [file pone.0008492.s003.tif]

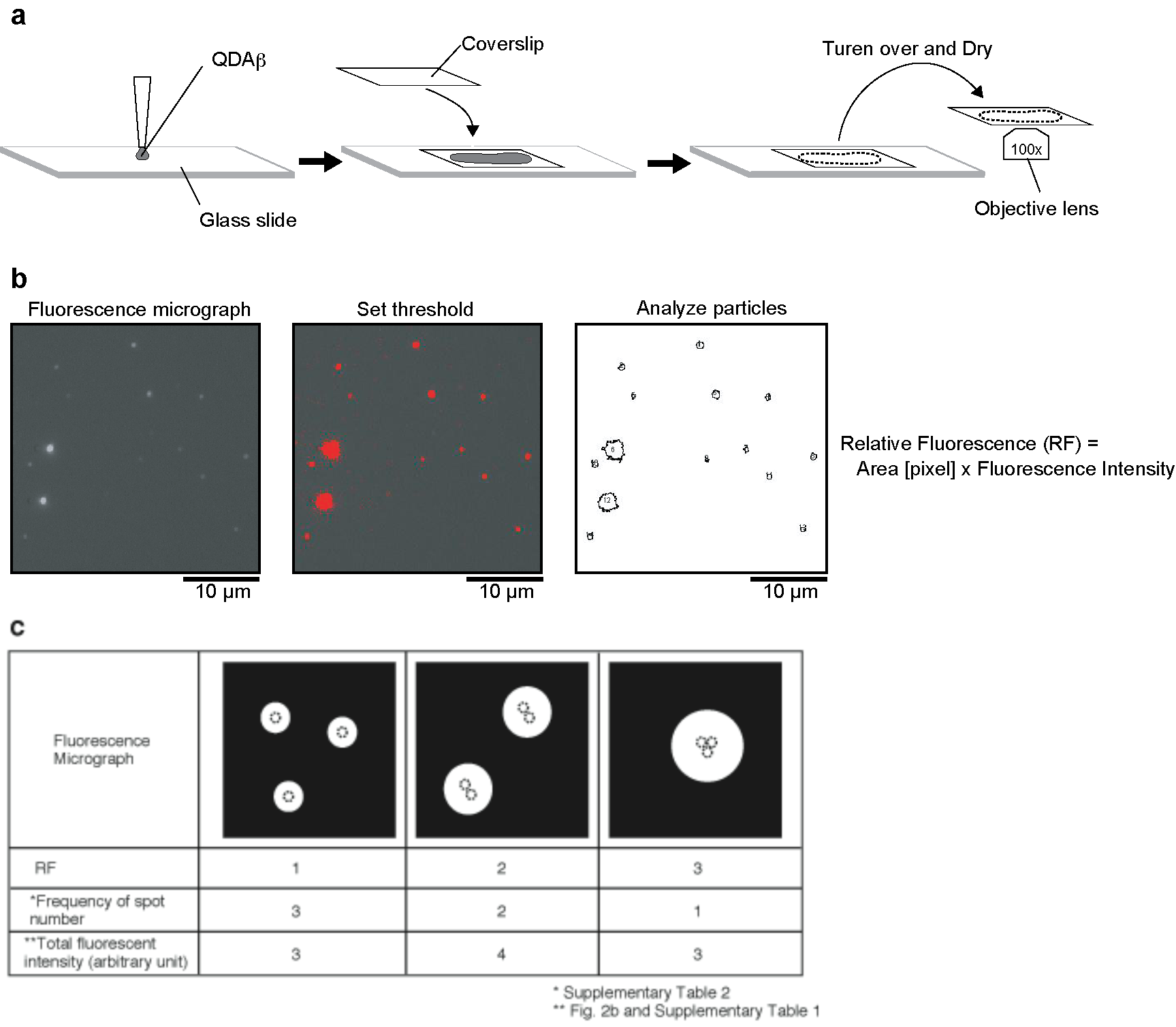

Supplement: Figure S2 — Analysis of fluorescence spots of QDAβ oligomers. (a) Preparation of samples. The coverslips for wide-field fluorescence microscopy observation were prepared by the modified method of Agrawal et al. [3]. An aliquot (2 µl) of oligomer sample solution, which was diluted to 1–10 nM, was spread between the glass slide and the coverslip. The coverslip was taken off, dried, and placed on a wide-field fluorescence microscope. The gray images (2040 pixel×1536 pixel: 175 µm×132 µm) were obtained using a 100x objective lens with a QD filter set. A micrograph represented an average of 5 frames (each exposure time was 0.2 s). (b) Measurement of relative fluorescence. The micrographs were analyzed using ImageJ software (NIH). In this analysis, we used a 1000×1000 pixel area in the central region of the micrographs because of aberration at the periphery. The micrographs were thresholded under the same conditions and then were analyzed using the “analyze particles” program of ImageJ. Relative fluorescence (RF) was defined as the product of the area size (pixel) and mean fluorescence intensity. The average RF of unlabeled QD-PEG-NH2 was expressed as 1 RF unit (RF1). In this study, ≤1.5, 1.5–2.5, 2.5–3.5, 3.5–4.5, ≥4.5 of RF were indicated as RF≤1, RF2, RF3, RF4, and RF≥5, respectively. Each analysis averaged 10 micrographs (one micrograph contained several hundred particles). (c) Frequency of spot number and total fluorescent intensity. Spot number (Table S2) and total fluorescent intensity (Figure 2b and Table S1) reflect the number of oligomers and the number of QDs belonging to each RF class. [3] Agrawal A, Deo R, Wang GD, Wang MD, Nie S (2008) Nanometer-scale mapping and single-molecule detection with color-coded nanoparticle probes. Proc Natl Acad Sci U S A 105: 3298–3303. (0.43 MB TIF) [file pone.0008492.s004.tif]

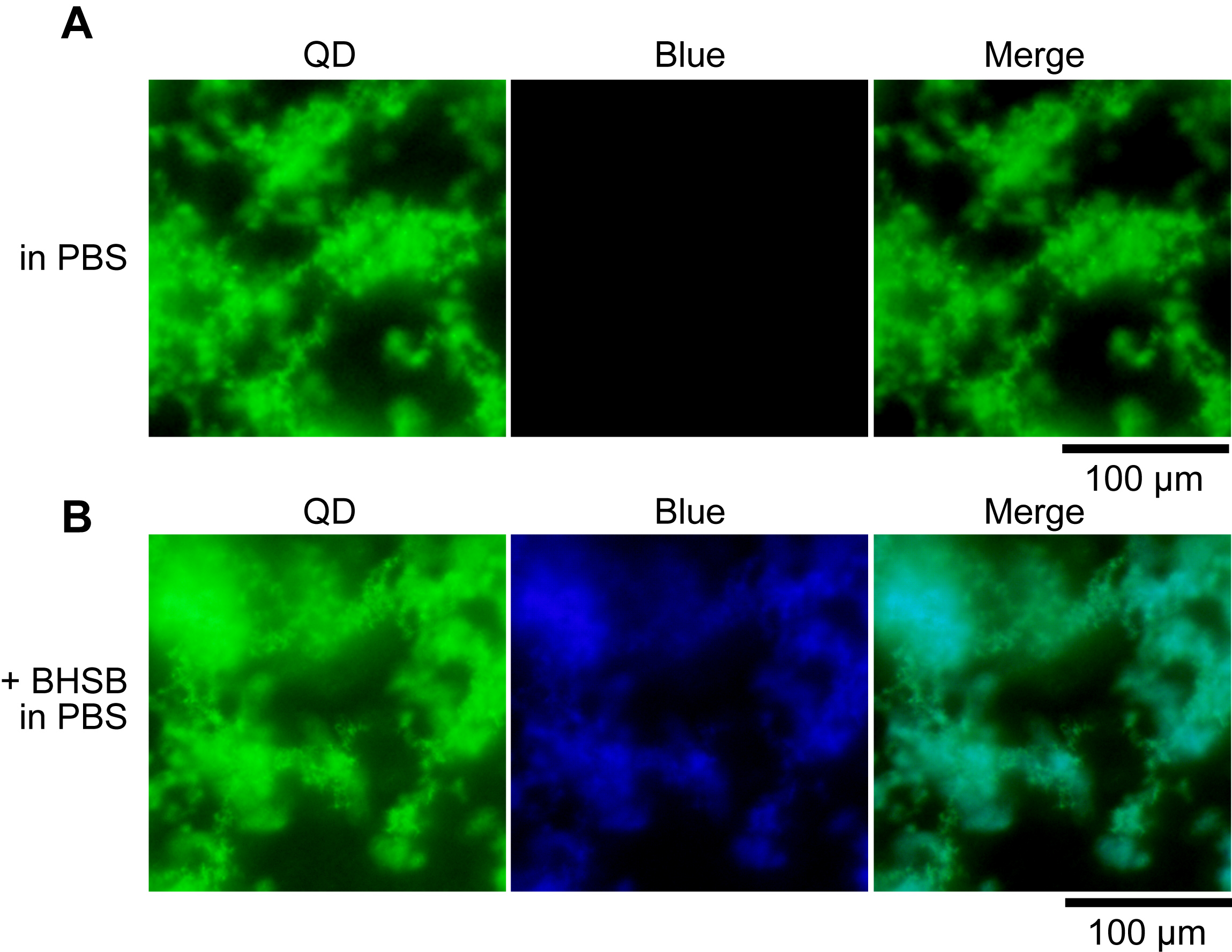

Supplement: Figure S3 — Staining of Aβ coaggregates by FSB derivative, (E,E)-1,4-bis(4-hydroxy)styrylbenzene. 0.1% QDAβ(6)-containing Aβ42 (final concentrations: 50 nM QDAβ(6) and 50 µM Aβ42) were incubated in PBS without (a) or with (b) 1 µM FSB derivative (BHSB) for 1 day at 37°C in 96 well glass bottom plates (MatTek). The aggregates were observed by wide-field fluorescence microscopy using a 20x objective lens with FITC (QD) or Blue (Blue) filter sets. Since the FSB derivative binds to the β-sheet structure of Aβ fibrils [4], it is likely that these aggregates are typical Aβ fibrils containing β-sheet structure. [4] Flaherty DP, Walsh SM, Kiyota T, Dong Y, Ikezu T, et al. (2007) Polyfluorinated bis-styrylbenzene beta-amyloid plaque binding ligands. J Med Chem 50: 4986–4992. (2.49 MB TIF) [file pone.0008492.s005.tif]

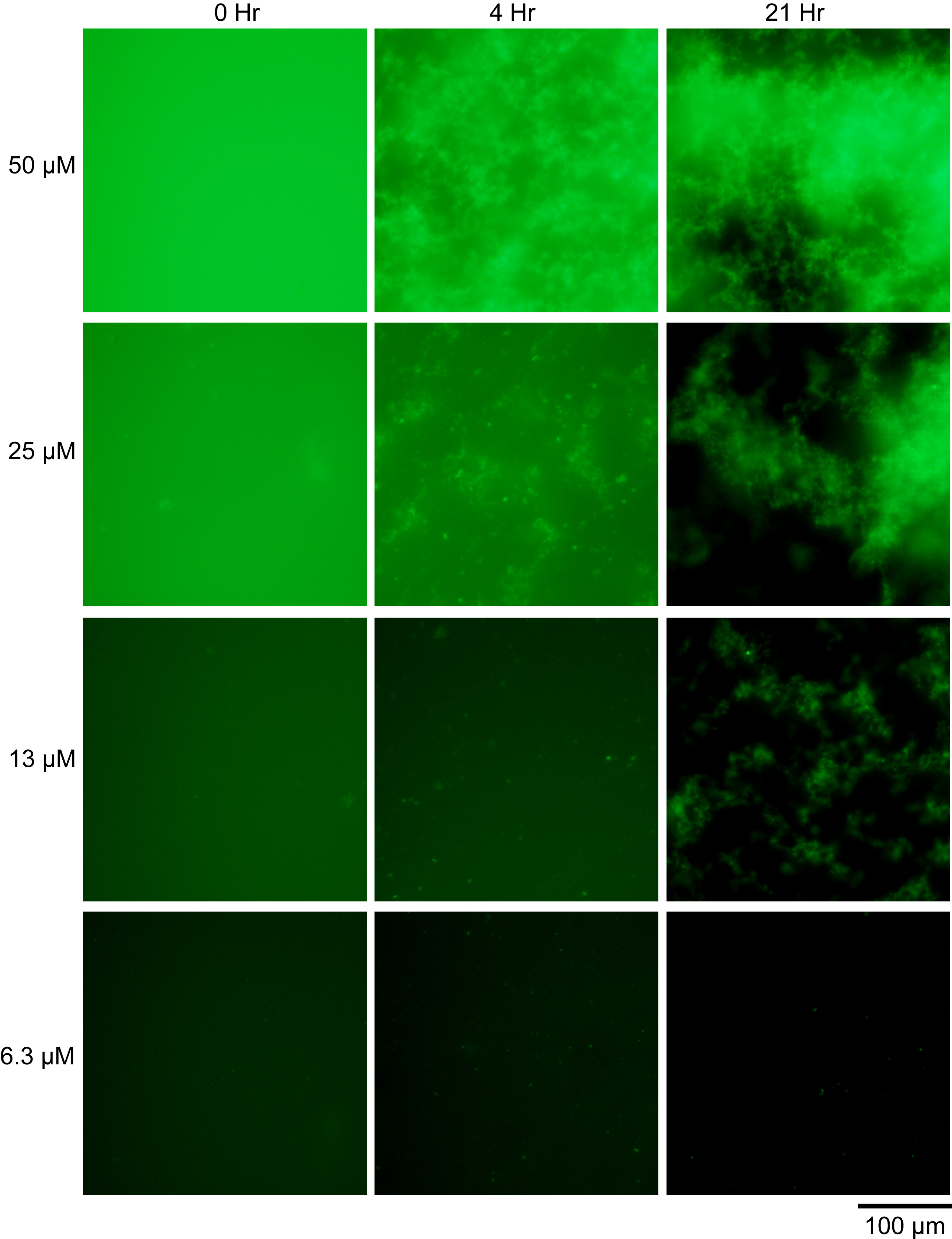

Supplement: Figure S4 — Dose- and time-dependent coaggregation. 0.1% QDAβ(6)-containing Aβ42 (50, 25, 13, and 6.3 µM of Aβ42) were incubated at 37°C in 96 well glass bottom plates. The samples were observed at 0, 4, and 21 h from the start of incubation by wide-field fluorescence microscopy using a 20x objective lens with FITC filter set. No aggregates were observed in all 0 h samples. Although dose- and time-dependent aggregation were observed in the 50, 25, and 13 µM samples, aggregates were not observed in the 6.3 µM sample, suggesting that the critical concentration for Aβ42 aggregation was between 6.3–13 µM under these conditions. (7.12 MB TIF) [file pone.0008492.s006.tif]

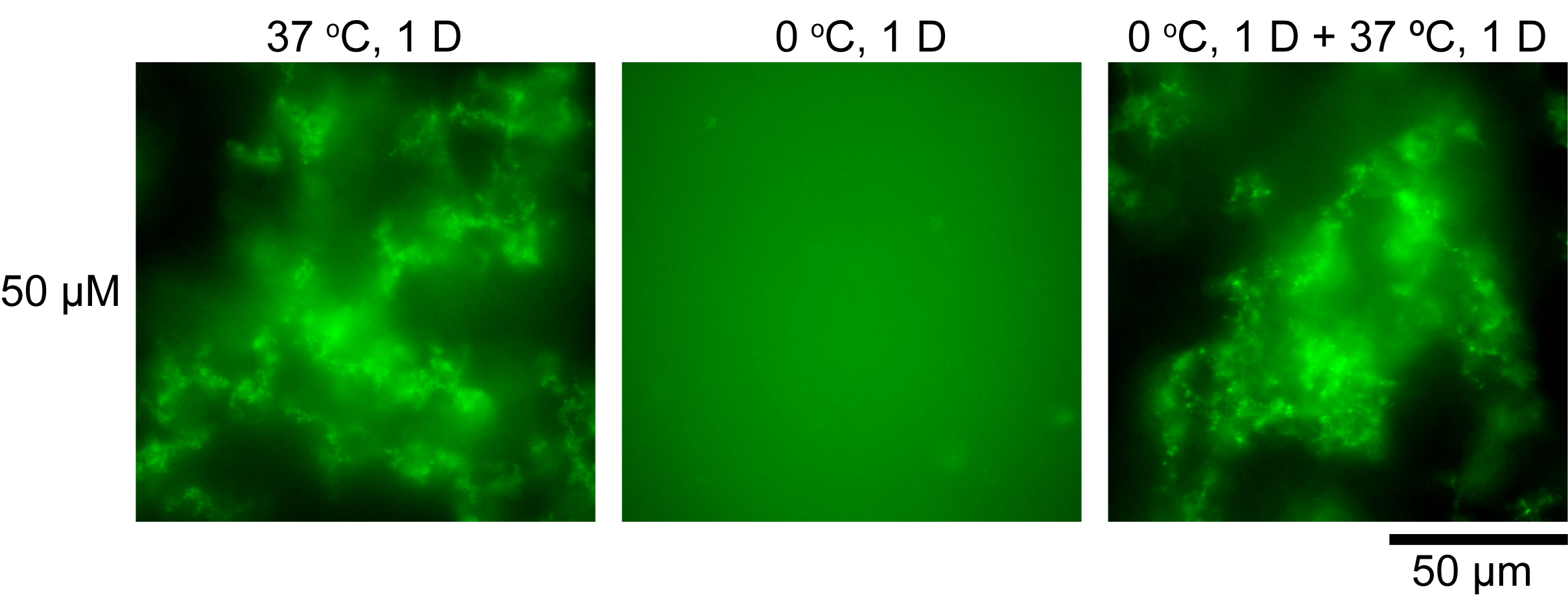

Supplement: Figure S5 — Temperature-dependent Aβ aggregation. 0.1% QDAβ(6)-containing Aβ42 (final concentration 50 µM) was incubated for 1 day at 37°C (left), for 1 day on ice (middle), and for 1 day at 37°C after 1 day on ice (right), and observed by wide-field fluorescence microscopy using a 100x objective lens with FITC filter set. The results showed that Aβ aggregates were not formed after 1 day at 0°C incubation (middle). The sample on ice formed aggregates by additional incubation (right), suggesting that the 0.1% QDAβ(6)-containing Aβ42 mixture can be stored on ice for at least 1 day. (1.67 MB TIF) [file pone.0008492.s007.tif]

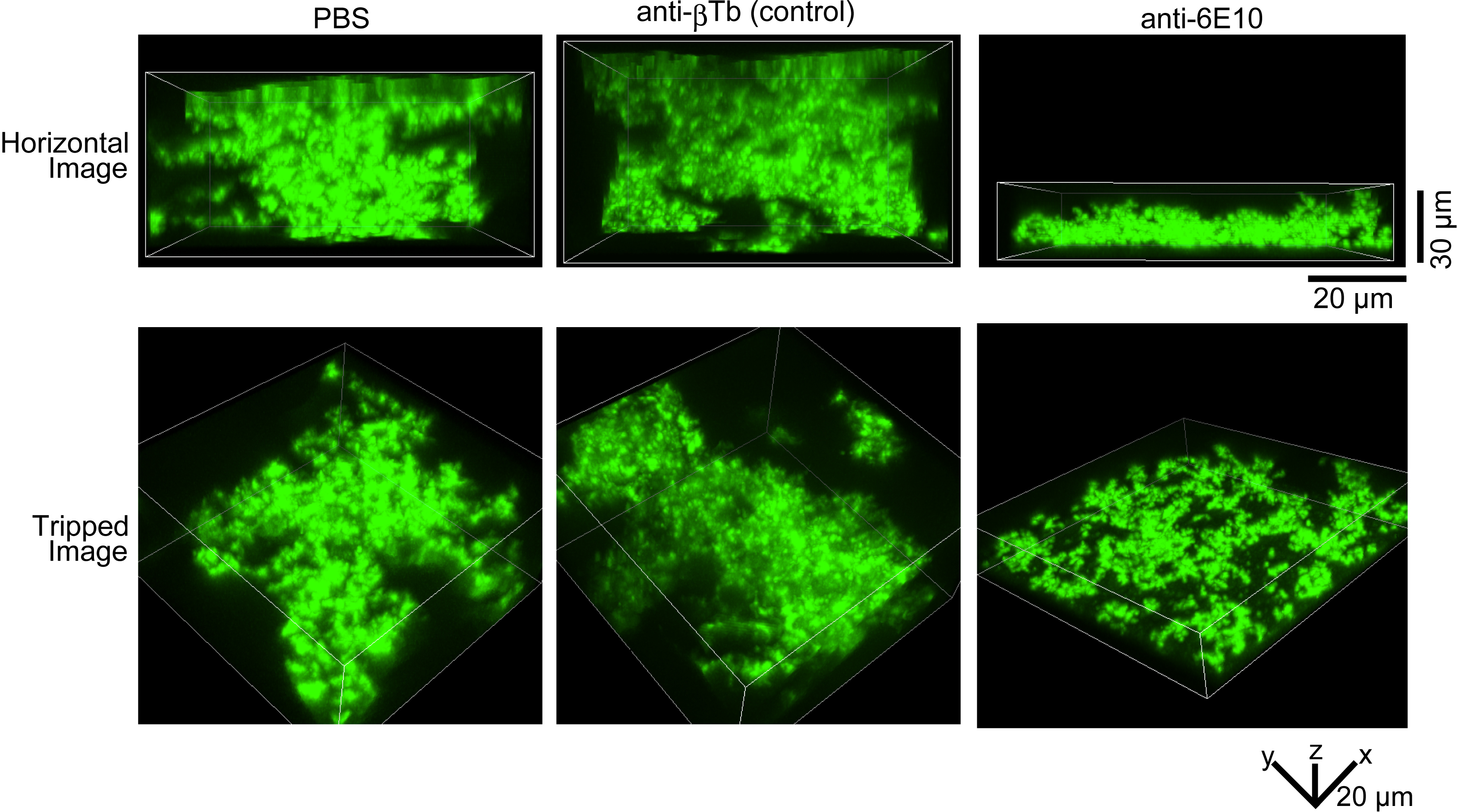

Supplement: Figure S6 — 3D reconstruction images of Aβ aggregation inhibition by anti-Aβ antibody. 0.1% QDAβ(6)-containing Aβ42 (final concentration 13 µM) was incubated in PBS without antibody (left), with anti-βTubulin (anti-βTb) antibody (middle), and with anti-6E10 antibody (right) for 1 day at 37°C in 96 well glass bottom plates, and observed by swept-field laser-scanning confocal microscopy using a 488 nm excitation laser (75%) and a 100x objective lens. The movies of these 3D images are supplied in Movie S2-S4. (3.87 MB TIF) [file pone.0008492.s008.tif]

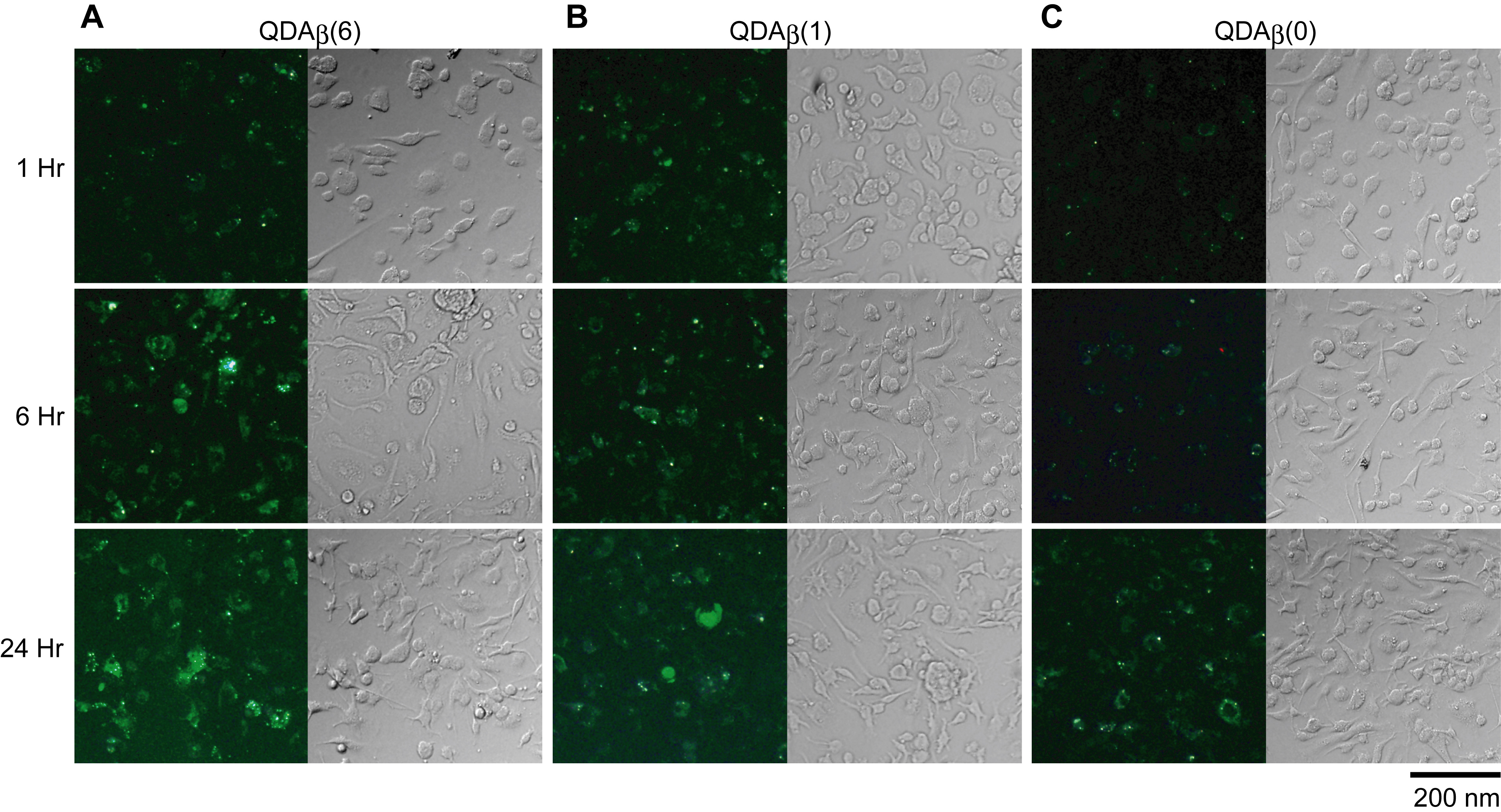

Supplement: Figure S7 — Microglial uptake of monomeric QDAβ(6), QDAβ(1), and QDAβ(0). Medium containing monomeric QDAβ(6) (a), QDAβ(1) (b), or QDAβ(0) (c) (final concentration 50 nM) was added to primary cultured mouse microglia in 96-well glass bottom plates (50,000 cells/well), and incubated for 1, 6, and 24 h time periods. The cells were fixed with 4% PFA and observed by wide-field fluorescence microscopy using a 20x objective lens with QD filter set. (8.75 MB TIF) [file pone.0008492.s009.tif]

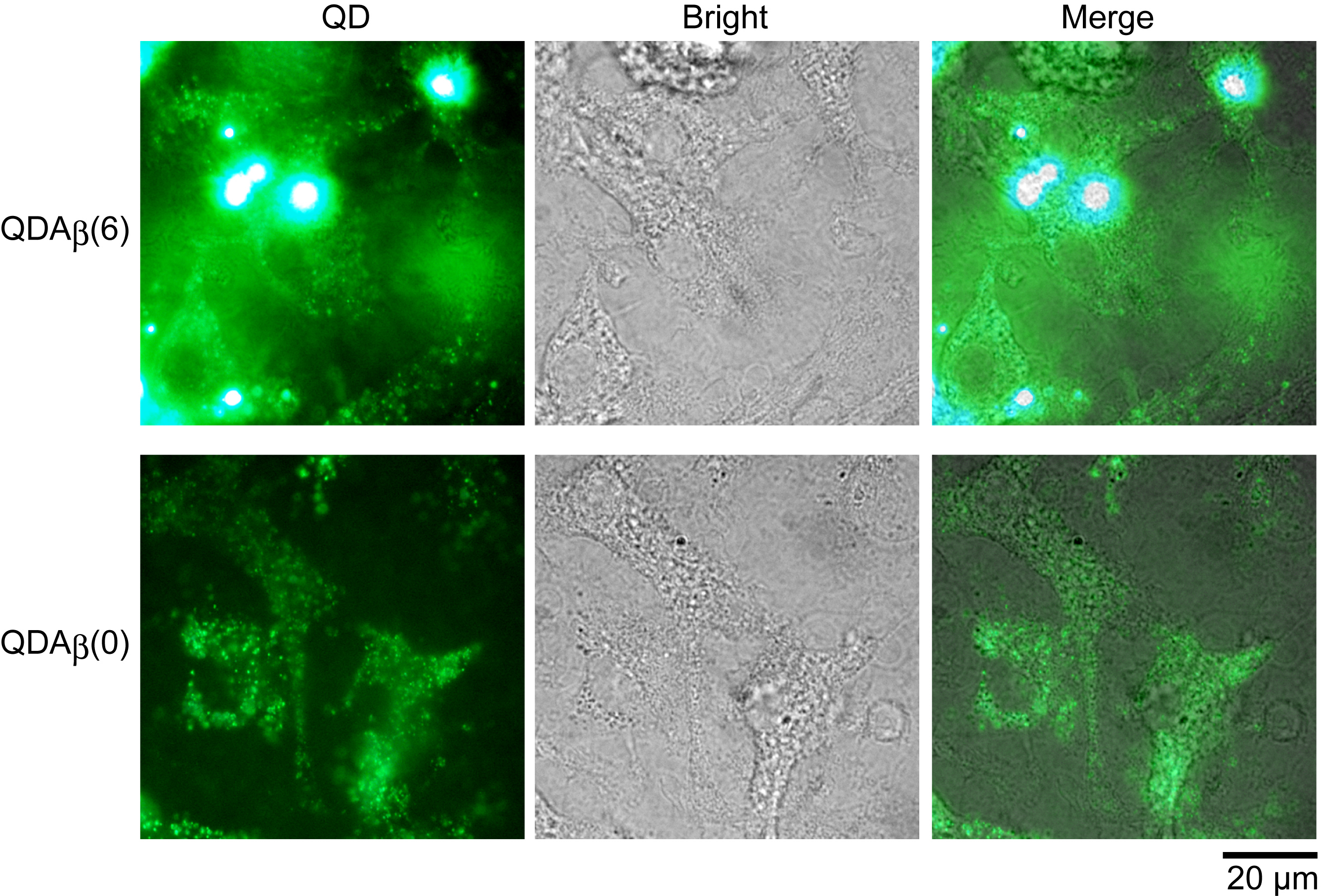

Supplement: Figure S8 — Magnified observation of microglia with ingested monomeric QDAβ(6) and QDAβ(0). Primary mouse microglia were incubated with monomeric QDAβ(6) or QDAβ(0) (final concentration 50 nM) for 24 h, followed by fixation with 4% PFA, and observed by wide-field fluorescence microscopy using a 100x oil objective lens (TE-300, Nikon Instruments) and QD filter set (green). (5.75 MB TIF) [file pone.0008492.s010.tif]

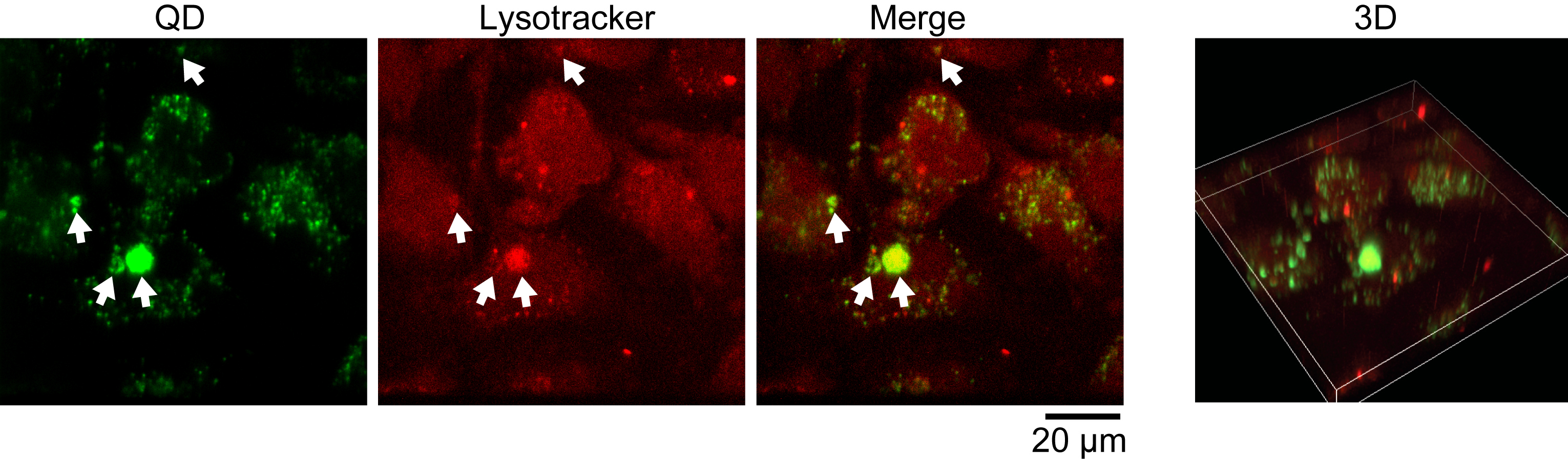

Supplement: Figure S9 — Co-localization of ingested QDAβ6) and Lysotracker in microglia. Primary mouse microglia were incubated with 50 nM monomeric QDAβ6) for 24 h, followed by incubation with 50 µM Lysotracker for an additional 30 min. The cells were fixed with 4% PFA, and observed by Swept-field laser-scanning confocal microscopy using 488 nm excitation (QD, green) and 568 nm excitation (Lysotracker, red). Far right panel is the 3D reconstruction image in the same field. The movie of this 3D image is in Movie S5. (3.22 MB TIF) [file pone.0008492.s011.tif]
