# Supplementary material for: Real-Time Imaging and Quantification of Amyloid-β Peptide Aggregates by Novel Quantum-Dot Nanoprobes
Source: PLoS One. 2009 Dec 30;4(12):e8492. doi: 10.1371/journal.pone.0008492 (PMC2794548; doi:10.1371/journal.pone.0008492)
Supplement: Supplementary file 2 [file pone.0008492.s001.doc]

a

Samples / RF	QD-PEG-NH_2 50 mM Borate (4 ºC)		QDAß(6) Water (0 ºC, 3W)		QDAß(6) PBS (4 ºC, 6 W)		QDAß(6) SDS, PBS (37 ºC, 3W)	
	Mean	SD	Mean	SD	Mean	SD	Mean	SD
≤1(<1.5)	80.9	10.9	76.2	7.1	29.7	9.5	26.1	4.9
2 (1.5–2.5)	15.8	9.5	15.8	6.0	21.6	5.5	18.5	4.3
3 (2.5–3.5)	2.2	1.0	5.1	2.6	13.2	4.5	11.9	2.4
4 (3.5–4.5)	0.8	1.3	0.7	1.0	5.6	3.1	7.7	2.8
≥5 (>4.5)	0.2	0.7	2.2	3.6	29.9	15.2	35.7	9.8

b

Samples / RF	QDAß(0) SDS, PBS (37 ºC, 1D)		QDAß(1) SDS, PBS (37 ºC, 1D)		QDAß(6) SDS, PBS (37 ºC, 1D)	
	Mean	SD	Mean	SD	Mean	SD
≤1(<1.5)	62.6	13.0	47.4	8.4	29.1	7.0
2 (1.5–2.5)	20.8	5.6	23.8	1.9	17.1	3.2
3 (2.5–3.5)	7.3	4.2	11.0	3.7	10.2	3.6
4 (3.5–4.5)	3.8	3.1	6.3	2.9	7.6	1.8
≥5 (>4.5)	5.4	3.6	11.4	7.0	35.9	9.3

c

Samples / RF	QDAß(0) PBS (37 ºC, 1D)		QDAß(1) PBS (37 ºC, 1D)		QDAß(6) PBS (37 ºC, 1D)	
	Mean	SD	Mean	SD	Mean	SD
≤1(<1.5)	69.8	9.9	61.7	12.1	60.2	6.3
2 (1.5–2.5)	19.9	5.5	20.9	4.7	23.4	4.2
3 (2.5–3.5)	6.4	3.1	8.8	2.6	8.0	2.8
4 (3.5–4.5)	1.5	1.8	3.4	2.6	3.0	2.7
≥5 (>4.5)	2.4	2.3	5.2	4.9	5.4	3.8
