# Supplementary material for: Real-Time Imaging and Quantification of Amyloid-β Peptide Aggregates by Novel Quantum-Dot Nanoprobes
Source: PLoS One. 2009 Dec 30;4(12):e8492. doi: 10.1371/journal.pone.0008492 (PMC2794548; doi:10.1371/journal.pone.0008492)
Supplement: Supplementary file 3 [file pone.0008492.s002.doc]

a (EM)

RF	1	2	3	4	≥5
(1) No-incubation	90.3	7.6	1.4	0.3	0.4
(2) Incubation (+SDS)	63.1	16.3	6.3	3.6	7.5
(2) - (1)	(76)	8.7	4.9	3.3	7.1

b (AFM)

Multimerization	1-mer	2-mer	3-mer	4-mer	≥5-mer
Incubation (+SDS)	82	8.1	4.8	2.4	2.4
